# Impact of Pressurized Intraperitoneal Aerosol Chemotherapy on Quality of Life and Symptoms in Patients with Peritoneal Carcinomatosis: A Retrospective Cohort Study

**DOI:** 10.1155/2017/4596176

**Published:** 2017-02-21

**Authors:** Hugo Teixeira Farinha, Fabian Grass, Amaniel Kefleyesus, Chahin Achtari, Benoit Romain, Michael Montemurro, Nicolas Demartines, Martin Hübner

**Affiliations:** ^1^Department of Visceral Surgery, University Hospital of Lausanne (CHUV), Lausanne, Switzerland; ^2^Department of Gynecology, University Hospital of Lausanne (CHUV), Lausanne, Switzerland; ^3^Department of Digestive Surgery, Strasbourg University, Strasbourg, France; ^4^Department of Oncology, University Hospital of Lausanne (CHUV), Lausanne, Switzerland

## Abstract

*Background*. Peritoneal cancer treatment aims to prolong survival, but preserving Quality of Life (QoL) under treatment is also a priority. Pressurized Intraperitoneal Aerosol Chemotherapy (PIPAC) is a novel minimally invasive repeatable treatment modality. The aim of the present study was to assess QoL in our cohort of PIPAC patients.* Methods*. Analysis of all consecutive patients included from the start of PIPAC program (January 2015). QoL (0–100: optimal) and symptoms (no symptom: 0–100) were measured prospectively before and after every PIPAC procedure using EORTC QLQ-C30.* Results*. Forty-two patients (M : F = 8 : 34, median age 66 (59–73) years) had 91 PIPAC procedures in total (1 : 4x, 17 : 3x, 12 : 2x, and 12 : 1x). Before first PIPAC, baseline QoL was measured as median of 66 ± 2.64. Prominent complaints were fatigue (32 ± 4.3) and digestive symptoms as diarrhea (17 ± 3.75), constipation (17 ± 4.13), and nausea (7 ± 2.54). Overall Quality of Life was 64 ± 3.75 after PIPAC#1 (*p* = 0.57), 61 ± 4.76 after PIPAC#2 (*p* = 0.89), and 70 ± 6.67 after PIPAC#3 (*p* = 0.58). Fatigue symptom score was 44 ± 4.86 after PIPAC#1 and 47 ± 6.69 and 34 ± 7.85 after second and third applications, respectively (*p* = 0.40). Diarrhea (*p* = 0.31), constipation (*p* = 0.76), and nausea (*p* = 0.66) did not change significantly under PIPAC treatment.* Conclusion*. PIPAC treatment of peritoneal carcinomatosis had no negative impact on patients' overall QoL and its components or on main symptoms. This study was registered online on Research Registry (UIN: 1608).

## 1. Introduction

Requirements for optimal oncological treatment include oncological efficacy (tumor response, survival) but also low toxicity, few side effects, and no negative impact on Quality of Life (QoL). Peritoneal carcinomatosis (PC) remains a particular challenge with sparse treatment options but high potential for side effects and complications [1, 2].

The effect of systemic chemotherapy remains limited on the peritoneum due to low penetration and relative resistance of peritoneal nodules. Combining several active agents has increased efficacy but was also associated with considerable risk for side effects with negative impact on QoL [3]. Hyperthermic Intraperitoneal Chemotherapy (HIPEC) overcomes some of the pharmacokinetic limitations and improves survival in selected patients [4, 5] at the price of high morbimortality and a negative impact on QoL for months after the procedure [6].

Intraperitoneal Aerosol Chemotherapy (PIPAC) was developed to disperse the active agents inside the peritoneal cavity by laparoscopy without tumor debulking, thus allowing for repeated treatments [7–9]. The few available studies reported encouraging objective tumor response of 50–86% and only little toxicity in patients with therapy-refractory PC of various origins [10–12]. Only one study reported on QoL under PIPAC treatment so far [13].

The present analysis was performed to evaluate QoL and symptoms under PIPAC treatment in a consecutive cohort of patients with PC.

## 2. Methods

This study included all consecutive patients treated by PIPAC from January 2015 until April 2016. All patients were discussed in the setting of a multidisciplinary tumor board to determine the best treatment option for each patient. PIPAC was considered only in patients with chemoresistant isolated peritoneal carcinomatosis who were not eligible for cytoreductive surgery (CRS) and Hyperthermic Intraperitoneal Chemotherapy (HIPEC) due to medical or surgical contraindications. The study was approved by the Institutional Review Board (Number 2016-00274) and all patients provided written consent prior to surgery. STROBE criteria (http://strobe-statement.org/) were followed for reporting of the study (http://www.researchregistry.com/; UIN: 1608).

### 2.1. PIPAC Procedure and Treatment Algorithm

**Figure 1:**
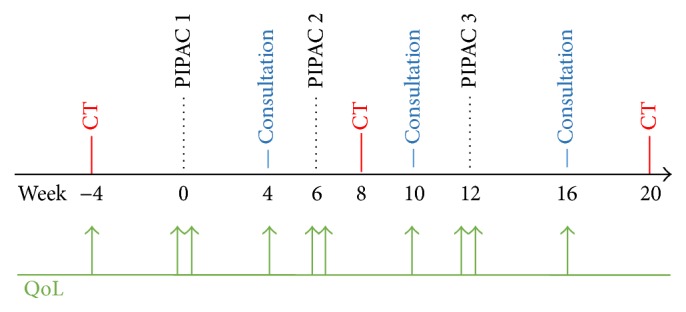
Treatment algorithm for Pressurized Intraperitoneal Aerosol Chemotherapy (PIPAC). PIPAC treatment was scheduled as repeated application (3x) at 6-week intervals. Thoracoabdominal computed tomography (CT) was performed 4 weeks prior to PIPAC#1, between PIPAC#2 and PIPAC#3, and after the completion of intraperitoneal treatment to search for extraperitoneal disease. Quality of Life (QoL) was systematically assessed (EORTC QLQ-C30) during every patient encounter: in outpatient consultation, before surgery, and at discharge.

Surgical setup, treatment regimens, and safety checklist were adopted from recommendations by Solaß et al. [9, 17]. Three PIPAC treatments were scheduled at 6-week intervals upon decision of the multidisciplinary tumor board (Figure 1). Systematically, thoracoabdominal computed tomography (CT) was performed not exceeding four weeks prior to first PIPAC and between PIPAC#2 and PIPAC#3 to actively rule out extraperitoneal disease. A third CT was scheduled 2 months after the completed 3 PIPAC cycles. Every patient was seen in outpatient consultation 4 weeks after PIPAC treatment for monitoring of complications and evaluation of contraindications to proceed with further PIPAC. QoL assessment was performed before surgery, at discharge, and every time when the patient was seen in outpatient consultation (Figure 1).

### 2.2. Assessment of Quality of Life and Symptoms

European Organization for Research and Treatment of Cancer (EORTC) generic questionnaire QLQ-C30 (version 3.0) was used to measure QoL and symptoms [14, 18]. QLQ-C30 is a 30-question self-administered questionnaire inquiring about global health status, 9 individual symptoms, and 5 functional scales. Validated versions were provided in French, English, Italian, and German. The EORTC QLQ-C30 scoring manual was followed in terms of scoring QoL data and with regard to missing answers [19]. For statistical analysis, the 30 scores were linearly converted to a 0–100 scale according to EORTC recommendations [19]. Of note, high functional scores indicate a high level of function (optimum: 100), while high symptom scores represented high degree of symptoms (optimum: 0). A mean difference of ±5 was considered to be of no clinical relevance for the patient, while ±5–10 and ±10–20 points represented small and modest clinical differences, respectively [15].

### 2.3. Data Management

Pertinent demographic and surgical data was prospectively recorded in coded form in a computerized data base (secuTrial®, IAS GmbH, Berlin). Performance status was assessed according to the Eastern Cooperative Oncology Group (ECOG) [20]. Intraoperative data included peritoneal cancer index (PCI) [16], ascites (mL), adhesiolysis, and operative time (min). Postoperative hospital stay and 30-day complications (Clavien classification [21]) were reported.

### 2.4. Predefined Subgroup Analyses

Overall QoL under PIPAC treatment was compared between patients with PC of gynecological versus digestive origin to detect potential differences between those different patient groups and entities.

Reaction to treatment and side effects might be different after first application as compared with consecutive administration. Further, cumulative toxicity might decrease tolerance to repeated application. Therefore, QoL and symptoms were analyzed separately for PIPAC#1 as compared to repeated PIPAC procedures.

A possible direct relationship was assessed between higher intraperitoneal tumor load (measured by PCI) and preoperative QoL. And finally, the hypothesis has tested whether low baseline QoL was correlated with longer hospital stay after PIPAC treatment.

### 2.5. Statistical Analysis and Interpretation

Continuous variables were presented as mean with standard error of the mean (SEM) or median with range or interquartile range (IQR) as appropriate. Student's *t*-test and Mann–Whitney *U* test were used for statistical comparisons depending on the distribution. Categorical variables were reported as frequencies (%) and compared with chi-square test. Statistical correlations were tested by use of Spearman's rank correlation. A *p* value <0.05 was considered to be statistically significant in all tests. Statistical analyses were performed and figures were produced with SPSS v20 statistical software (Chicago, IL, USA) and GraphPad Prism 7 (GraphPad Software, Inc., La Jolla, CA, USA).

## 3. Results

Forty-two consecutive patients were included in the present analysis as detailed in Table 1. Indication was carcinomatosis of gynecological origin in 21 patients. The digestive group included 14 patients with PC of colorectal and 3 of gastric origin (1 each for small bowel, appendicular, pseudomyxoma, and mesothelioma). One out of forty-two patients (2%) had combined systemic chemotherapy. Overall, 91 PIPAC procedures were performed in 42 patients (18 : ≥3, 12 : 2, and 12 : 1). Overall complication rate was 8.8% and median hospital stay was 3 (range 1–20).

### 3.1. Quality of Life and Symptoms during PIPAC Treatment

**Figure 2:**
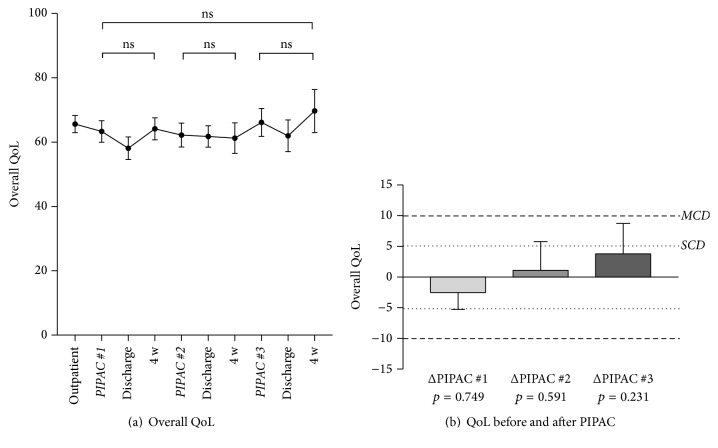
Quality of Life (QoL) under Pressurized Intraperitoneal Aerosol Chemotherapy (PIPAC) treatment. Overall Quality of Life (QoL: EORTC QLQ-C30 [14]) under PIPAC treatment is displayed (mean ± SEM). (a) No statistically significant difference (*p* < 0.05) was found when QoL was compared before and after different PIPAC applications (a, b). The dotted lines (b) represent the thresholds for small (SCD) and moderate clinically relevant differences (MCD), respectively [15].

**Figure 3:**
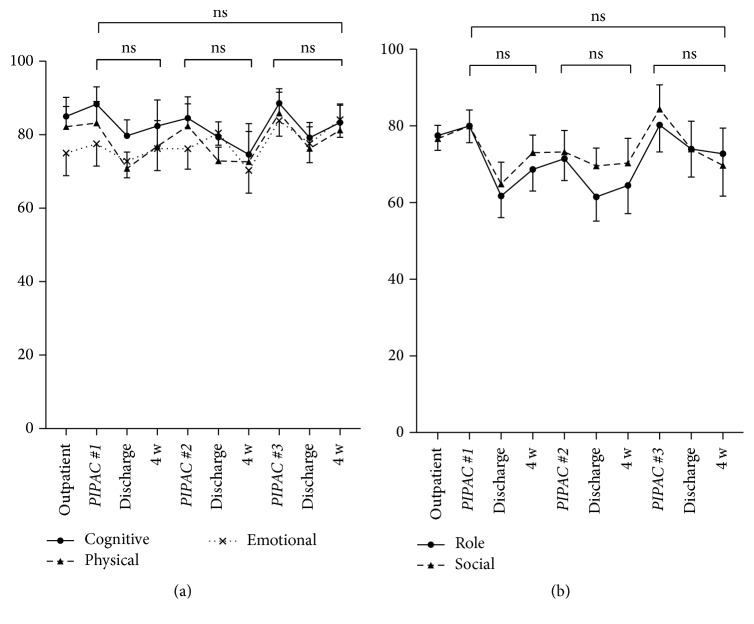
Cognitive, physical, emotional, role, and social functioning under Pressurized Intraperitoneal Aerosol Chemotherapy (PIPAC). Quality of Life (QoL: EORTC QLQ-C30 [14]) components under PIPAC treatment (mean ± SEM). No significant difference (*p* < 0.05) was found when QoL was compared before and after different PIPAC applications (ns—not significant).

Overall QoL score was 66 ± 2.6 at baseline before the start of PIPAC treatment. QoL scores during the treatments cycles are displayed in Figure 2(a). QoL was not significantly different before and after PIPAC#1, PIPAC#2, and PIPAC#3, respectively, and the threshold for small or moderate clinically relevant difference was not reached at any time point (Figure 2(b)). Similarly, no significant changes were noted under PIPAC treatment for the QoL components* cognitive, physical, emotional, role, and social functioning*; individual curves are provided as in Figure 3.

**Figure 4:**
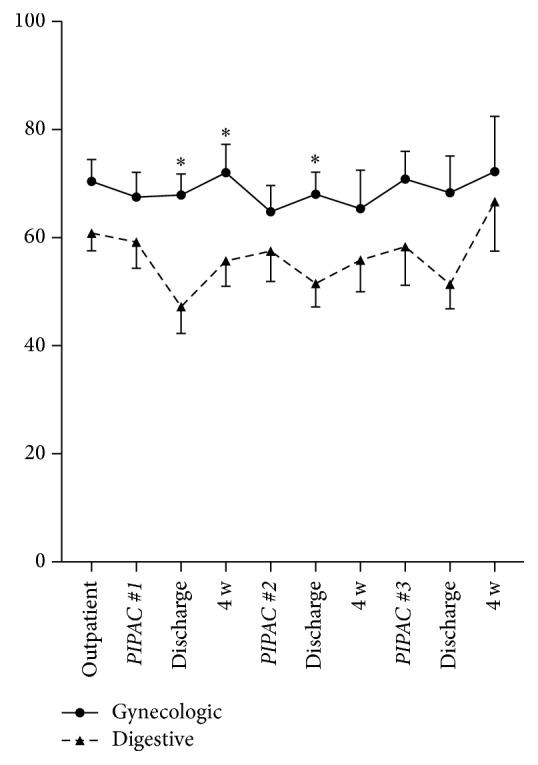
Quality of Life (QoL) under Pressurized Intraperitoneal Aerosol Chemotherapy (PIPAC) in patients with gynecological versus digestive malignancies. Overall Quality of Life (QoL: EORTC QLQ-C30 [14]) is displayed as mean ± SEM. *∗* indicates statistical significance (*p* < 0.05).

Overall QoL was separately analyzed in gynecological and digestive patients (Figure 4). The latter group tended to have lower scores throughout the treatment course with significant differences after PIPAC#1 (discharge: *p* = 0.03; 4 weeks: *p* = 0.02) and after PIPAC#2 (discharge: *p* = 0.01).

**Figure 5:**
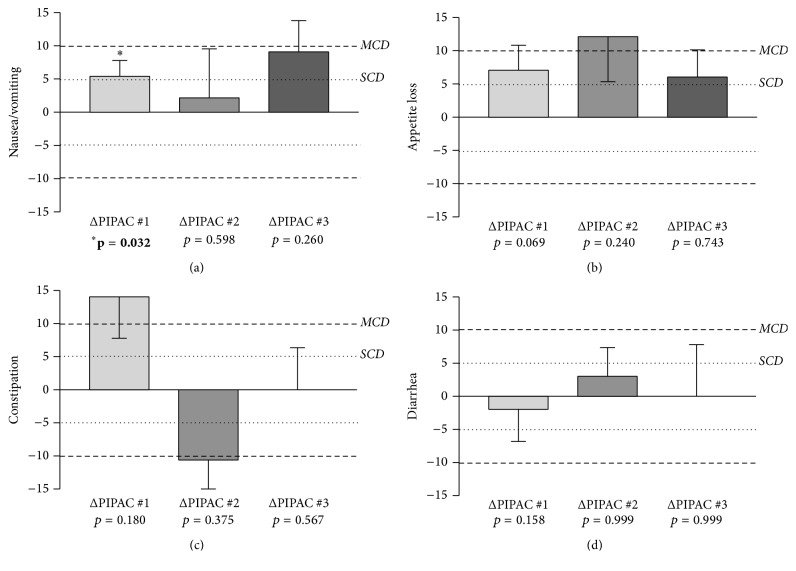
Digestive symptoms under Pressurized Intraperitoneal Aerosol Chemotherapy (PIPAC). Digestive symptoms were assessed by use of EORTC QLQ-C30 [14] and displayed as difference (Δ before − after). Statistical significance (*p* < 0.05) is highlighted (bold) and small (SCD) and modest (MCD) clinically relevant differences [15] are illustrated by dashed lines.

**Figure 6:**
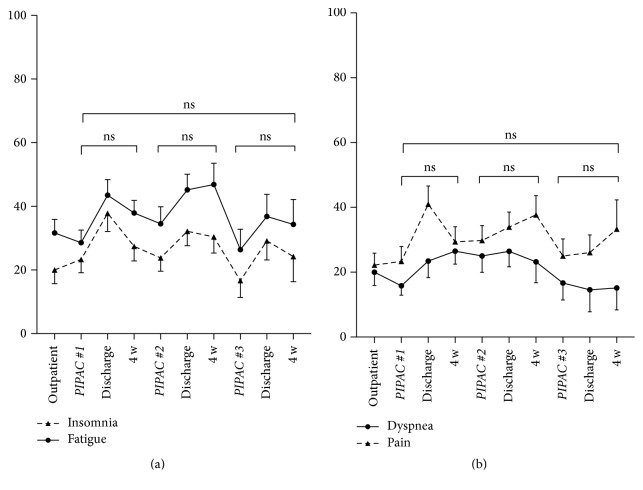
Nondigestive symptoms under Pressurized Intraperitoneal Aerosol Chemotherapy (PIPAC). Nondigestive symptoms (QoL: EORTC QLQ-C30 [14]) under PIPAC treatment (mean ± SEM). No significant difference (*p* < 0.05) was found when QoL was compared before and after different PIPAC applications (ns—not significant).

Prominent complaints at baseline were fatigue (32 ± 4.3) and the digestive symptoms diarrhea (17 ± 3.4), constipation (17 ± 4.1), and nausea (7 ± 2.5). Digestive symptoms before and after PIPAC are detailed for 1st, 2nd, and 3rd applications.* Nausea/vomiting* increased transitorily after PIPAC#1 (*p* = 0.03), just reaching the defined threshold for a small clinically relevant difference; no significant increase was present after PIPAC#2 and PIPAC#3, respectively (Figure 5(a)). For the symptoms* appetite loss, constipation, *and* diarrhea*, nonsignificant variation was noted in both directions (Figures 5(b), 5(c), and 5(d)). Nondigestive symptoms* insomnia, fatigue, pain, *and* dyspnea* did not show significant changes throughout PIPAC treatment (Figure 6).

### 3.2. QoL and Symptoms after First and Repeated PIPAC

**Figure 7:**
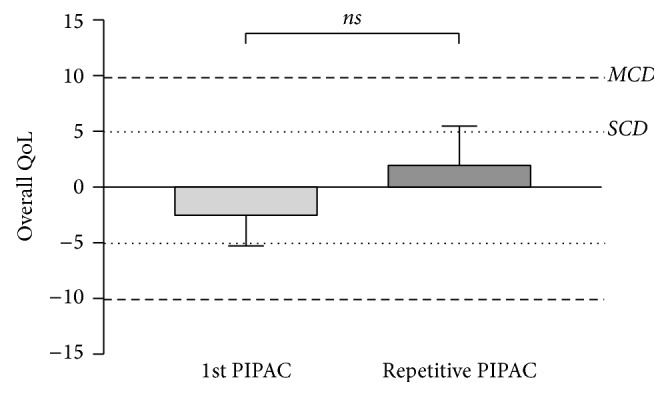
Quality of Life (QoL) change after Pressurized Intraperitoneal Aerosol Chemotherapy (PIPAC): 1st application versus repeated procedures. Quality of Life (QoL: EORTC QLQ-C30 [14]) as Δ before − after was compared for PIPAC#1 versus repeated procedures. Statistical significance: *p* < 0.05. ns—not significant. SCD: small clinically relevant difference, MCD: modest MCD clinically relevant difference [15].

**Figure 8:**
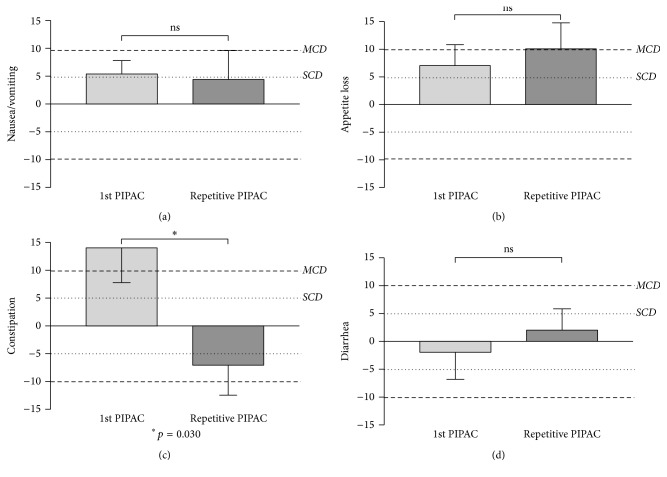
Symptom change after Pressurized Intraperitoneal Aerosol Chemotherapy (PIPAC): 1st application versus repeated procedures. Digestive symptoms were assessed by use of EORTC QLQ-C30 [14] and displayed as difference (Δ before − after). ^*∗*^Statistical significance (*p* < 0.05). SCD: small clinically relevant difference, MCD: moderate clinically relevant difference [15].

Change of overall QoL (Δ before − after) was small and nonsignificant for both 1st and repeated PIPAC procedures (*p* = 0.388) as shown in Figure 7. Performing similar analyses for digestive symptoms, there was a significantly higher symptom score for constipation after PIPAC#1 as compared to repeated PIPAC (*p* = 0.030), while no difference was measured with regard to nausea/vomiting, appetite loss, and diarrhea (Figures 8(a), 8(b), 8(c), and 8(d)).

### 3.3. Correlation between QoL, Tumor Load, and Hospital Stay (Figure 9)

There was no statistical correlation measured between intraperitoneal tumor load (PCI) and preoperative overall QoL (*ρ* = −0.169, *p* = 0.122). Higher preoperative QoL score was associated with shorter postoperative LoS (days) (*ρ* = −0.213, *p* = 0.05).

## 4. Discussion

PIPAC had no negative impact on patients' overall QoL and its components or on main symptoms. First and repetitive PIPAC application was equally well tolerated. Baseline QoL was good in patients with peritoneal disease and independent of intraperitoneal tumor load.

**Figure 10:**
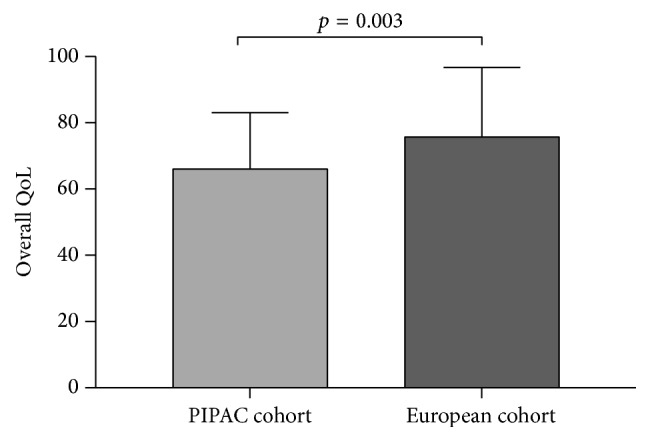
Overall Quality of Life (QoL) in PIPAC patients (Lausanne cohort) as compared with the general population. Overall QoL was compared by use of EORTC QLQ-C30 [14] for the Lausanne PIPAC cohort versus a European cohort of 16,151 healthy citizens (general population). The control group had a slightly better QoL score that was statistically significant but of small clinical relevance (<10%) according to Osoba et al. [15].

Overall QoL at baseline before start of PIPAC treatment was surprisingly high in the present cohort of patients with peritoneal carcinomatosis. As comparison, a control group of 16,151 healthy citizens had a significantly higher QoL score than this cohort (Figure 10) [22]. However, the observed absolute difference between both groups was marginal in terms of clinical relevance according to Osoba et al. [15]. Furthermore, there was no worse QoL in PIPAC patients with high intraperitoneal tumor load (assessed by PCI). The only difference was that patients with better overall QoL scores at baseline had a significant shorter hospital stay. These findings confirm two reports from the German PIPAC pioneer group. Tempfer reported in a phase II study on ovarian carcinomatosis patients a baseline global physical health score of 52.0 with improvements in QoL scores and symptoms under PIPAC treatment [11]. In the second study, Odendahl et al. assessed QoL and symptoms in a mixed cohort of PIPAC patients [13]. In the 48 reported patients with repeated PIPAC treatment, baseline global physical score was astonishingly high with 82 points. This score was not only higher than the baseline score of our own cohort but compared also favorably with the benchmark QoL scores of the general population cited above [22]. Under PIPAC treatment, QoL and symptoms remained unchanged in the Odendahl et al. study [13] as it was the case in our present series. There are two important differences between the two studies. First, 66 out of 114 eligible patients of the German cohort had to be excluded for missing QoL questionnaires [13]. QoL and symptoms of over the half of their cohort remain therefore unknown, while QoL assessment was complete in our present series. Second, QoL assessment was done only the day before surgery in the two German studies [11, 13]. Therefore, transitory deterioration might have been missed given the long washout period of about 6 weeks between PIPAC applications. For these reasons, in the present study, it was decided to add more time points to measure QoL and symptoms in between applications, namely, immediately at discharge day and 4 weeks after discharge. As suspected, some worsening of QoL and symptoms was observed at discharge (Figures 2(a) and 3), which was in most patients only 2-3 days after PIPAC treatment. However, those deviations from baseline were minimal and transitory.

It is important to assess QoL and symptoms under oncological treatment because both disease and treatment may have negative impact. A recent prospective multicenter longitudinal study evaluated results of systemic palliative chemotherapy in end-stage cancer patients. The authors observed no survival benefit and no improvements of QoL for patients with moderate and poor performance status (ECOG = 2, 3) at baseline. In patients with good baseline performance status (ECOG = 1), QoL was even significantly worse under systemic palliative chemotherapy [6]. In a secondary analysis, the authors compared patients receiving palliative chemotherapy versus best supportive care employing propensity score weighted adjustment. Patients under palliative systemic chemotherapy had a higher risk for cardiopulmonary resuscitation, invasive ventilation, and late hospice referrals. Patients without treatment had the same survival times but a significantly higher chance of dying in their preferred place instead of in the intensive care unit [23].

In the management of peritoneal carcinomatosis, HIPEC has a curative intent while PIPAC is considered palliation, since no data on long-term outcome after PIPAC is available so far. Therefore, their respective indications are different. In this context, it is important to weigh the expected survival benefits against morbid-mortality and impact on QoL. Two recent studies reported decreased QoL and increased symptoms 6–12 months after HIPEC treatment. And this was also observed in patients without postoperative complications [24, 25].

Good tolerance profile and QoL under PIPAC treatment allowed assessing bidirectional regimens combining systemic and intraperitoneal PIPAC treatment. Safety, tolerance, and QoL were still acceptable under bidirectional treatment that might offer a chance for downstaging and secondary CRS + HIPEC in selected patients [12, 26–28].

Concerning efficacy, first results of the pioneer center from Herne, Germany, were encouraging, but longer follow-up periods are required for assessment of oncological outcomes [11, 12, 28]. Furthermore, independent confirmation of histological regression under PIPAC treatment in a standardized way is needed. Future prospective studies should present histological results in a systematic way using a recently published tumor regression score [29].

Main limitations of this study are its small and heterogeneous patient cohort and its retrospective design. On the other hand, QoL assessment was prospectively performed in all consecutive patients from the beginning of the PIPAC program and no patient was excluded from this analysis. Furthermore, PIPAC procedure and treatment algorithms were standardized.

In summary, this study suggests that PIPAC had no negative impact on QoL in patients with peritoneal carcinomatosis. Digestive and nondigestive symptoms remained unchanged after a first PIPAC application but also after repeated treatments. Prospective evaluation of histological response rates and survival times under PIPAC treatment is underway.

## Figures and Tables

**Figure 9:**
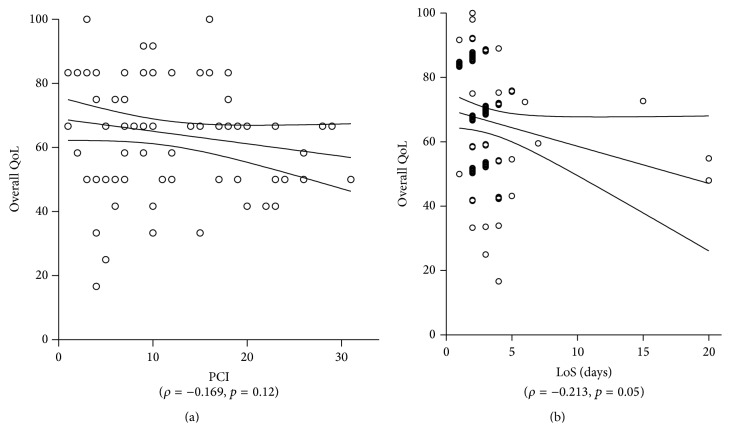
Quality of Life, tumor load, and hospital stay. Overall QoL was plotted against the extent of peritoneal disease (measured by the Peritoneal Cancer Index (PCI) [16]) (a) and length of hospital stay (LoS) (b).

**Table 1:** Baseline demographics and surgical details of patients treated with Pressurized Intraperitoneal Aerosol Chemotherapy (PIPAC).

		All patients (*n* = 42)	GYN (*n* = 21)	Digestive (*n* = 21)	*p* value
Demographics	Median age (years)	66 (59–73)	67 (63–74)	62 (55–72)	0.193
	Gender (male)	8 (19%)	—	8	—
	Median BMI (kg/m^2^)	22.5 (20–25)	23 (21–28)	22 (19–25)	**0.116**
	BMI < 18.5 kg/m^2^	2 (5%)	0	2	—
	ECOG (0-1)	36 (86%)	20 (95%)	76 (90%)	0.077

		All PIPAC (*n* = 91)	GYN (*n* = 51)	Digestive (*n* = 40)	*p* value

Intra-OP findings	PCI	10 (5–17)	9 (4–14)	15 (7–19)	**0.002**
	Ascites (mL)	50 (0–4000)	0 (0–300)	50 (0–4000)	**0.034**
	Adhesions	15 (16%)	9 (18%)	6 (15%)	0.735
	Operation time (min)	94 (89–108)	91 (87–97)	100 (92–117)	**0.002**

Median (IQR) or number of (%) as appropriate. Statistical significance (*p* < 0.05) is highlighted in bold.

BMI: Body Mass Index; ECOG: Eastern Cooperative Oncology Group performance status [20]; PCI: Peritoneal Cancer Index [16].

## Abbreviations

PC: Peritoneal carcinomatosis
QoL: Quality of Life
PIPAC: Pressurized Intraperitoneal Aerosol Chemotherapy
PCI: Peritoneal Carcinomatosis Index.
